# Chronic Ethanol Exposure: Pathogenesis of Pulmonary Disease and Dysfunction

**DOI:** 10.3390/biom5042840

**Published:** 2015-10-20

**Authors:** Nicole Traphagen, Zhi Tian, Diane Allen-Gipson

**Affiliations:** 1 Department of Pharmaceutical Sciences, College of Pharmacy, University of South Florida Health, Tampa, FL 33612, USA; E-Mails: ntraphagen@health.usf.edu (N.T.); ztian@health.usf.edu (Z.T.); 2 Department of Internal Medicine, Division of Allergy and Immunology, University of South Florida Health, Tampa, FL 33612, USA

**Keywords:** lung, ethanol, dysfunction, COPD, pneumonia

## Abstract

Ethanol (EtOH) is the world’s most commonly used drug, and has been widely recognized as a risk factor for developing lung disorders. Chronic EtOH exposure affects all of the organ systems in the body and increases the risk of developing pulmonary diseases such as acute lung injury and pneumonia, while exacerbating the symptoms and resulting in increased mortality in many other lung disorders. EtOH and its metabolites inhibit the immune response of alveolar macrophages (AMs), increase airway leakage, produce damaging reactive oxygen species (ROS), and disrupt the balance of antioxidants/oxidants within the lungs. In this article, we review the role of EtOH exposure in the pathogenesis and progression of pulmonary disease.

## 1. Introduction

Approximately 20 million people in the United States alone have met the criteria for alcoholism, which is a major problem worldwide and accounts for 3.8% of deaths globally [1,2,3]. Excessive EtOH use costs $24.6 billion in healthcare costs each year in the US, creating an enormous burden for our healthcare system [4]. In the United States alone, there are thousands of deaths occurring each year from EtOH-related diseases [5]. EtOH exposure and dependence produces chronic and toxic effects in many organs, including the lung. It has been well documented that chronic EtOH consumption can lead to extensive pulmonary dysfunction, including increased risk, severity of symptoms, and mortality in acute lung injury (ALI), pneumonia, and chronic obstructive pulmonary disease (COPD) [6,7,8]. To understand how chronic EtOH exposure impacts the progression of pulmonary diseases we need to gain a better understanding of EtOH-mediated dysregulation of the lung.

EtOH is primarily metabolized in the liver; however, some of the circulating EtOH reaches the lungs, where it diffuses from bronchial circulation, through the conducting epithelium, and into the conducting airways [9]. Upon reaching the airway, the EtOH is vaporized. Much of this vaporized EtOH is exhaled, but some of it dissolves a second time in the airway lining fluid, creating a damaging cycle of repeated exposure [9]. Furthermore, the metabolic products of EtOH can also damage the lungs [10,11]. The primary pathway for EtOH metabolism is through alcohol dehydrogenase (ADH), an enzyme that catalyzes the breakdown of EtOH into acetaldehyde in the liver and gastric mucosa. Acetaldehyde is further processed by aldehyde dehydrogenase (ALDH), which oxidizes the aldehyde to acetate [12]. However, when EtOH concentrations are consistently elevated, as with chronic EtOH exposure, it can also be metabolized through the cytochrome P450 2E1 (CYP2E1) into acetaldehyde. Part of the larger family of cytochrome p450 hemoprotein monooxygenases, CYP2E1 catalyzes the biotransformation of multiple drugs and toxins and is active in many tissues, including the lungs, where it metabolizes EtOH [10,13,14].

In the CYP2E1 pathway acetaldehyde is the major metabolite of EtOH, which has been linked to the generation of ROS and lipid peroxidation resulting in cellular oxidative stress [10]. This oxidative stress and disruption of signaling pathways has been implicated in the pathogenesis of many lung diseases, including ALI, asthma, and COPD. In addition to creating damaging oxidants EtOH impairs several important stages in the lung’s innate response to pathogens and injury, and disrupts the epithelial barrier, predisposing the lungs to bronchitis, pneumonia, and pulmonary edema [2,6].

## 2. EtOH Exposure Alters Airway Mucociliary Clearance

Mucociliary clearance is an important primary innate defense mechanism, which protects the lungs from deleterious effects of inhaled pollutants, allergens, and pathogens. It consists of three distinct components: the cilia, a mucus layer, and the airway surface liquid. Mucociliary dysfunction is common in chronic airway diseases, most notably in COPD [15]. Evidence revealed that chronic alcohol exposure increases COPD mortality, which is a leading cause of death in the US [7,16]. COPD is often associated with smoking, however in this review we focus solely on the effects of EtOH in the absence of cigarette smoke. It has also been well documented that EtOH is an independent risk factor for pneumonia, with chronic EtOH exposure increasing the risk for pneumonia eightfold [17]. EtOH affects ciliary beating in the airways, thus modulating mucociliary clearance of bacteria and pathogens [18,19]. Although the EtOH-mediated alterations in CBF were noted as early as the 1960s, the underlying mechanisms have only been recently elucidated [20]. Recent studies have further demonstrated the role of EtOH in the dysfunction of mucociliary clearance.

### 2.1. Acute EtOH Exposure Stimulates CBF

Acute EtOH exposure stimulates the beating of cilia in the airway through the endothelial nitric oxide synthase pathway (eNOS) [21]. In models of acute EtOH exposure, Simet *et al.* demonstrated that EtOH increases HSP90 phosphorylation at threonine residues [21]. Although HSP90 is primarily a protein-folding chaperone, when phosphorylated it can also function as a signal transducer [22]. In this study, they demonstrated that activated HSP90 increases colocalization with eNOS, which both translocated to the axoneme and resulted in stimulated ciliary beat frequency (CBF). However, this effect can be reversed using drugs in the benzoquinone ansamycin class, which have been shown to specifically bind and inhibit HSP90 [21,23]. In models of acute EtOH exposure, treatment with the benzoquinone ansamycin drug geldanamycin blocked the colocalization of HSP90 with eNOS and the subsequent stimulation of CBF was inhibited [21,23]. Acute EtOH exposure has also been shown to improve lung function in adults. Siu *et al.* determined that alcohol, independent of any other risk factors such as smoking and heart disease, had better 1-second forced expiratory volume (FEV1), forced vital capacity (FVC), and FEV1/FVC values, all of which measure respiratory function [24].

### 2.2. Chronic EtOH Exposure Desensitizes Ciliary Response

The effects of chronic EtOH over-exposure are drastically different from the effects of short-term exposure. Reduced bacterial clearance in the lungs of ethanol-fed animals was first noted as early as 1964 [25]. This reduced bacterial clearance is a common disorder in individuals with a history of EtOH consumption, known as EtOH-induced ciliary dysfunction (AICD). AICD is characterized by desensitized cyclic nucleotide dependent kinases, which regulate phosphorylation and mediate ciliary beating [19]. In contrast to short-term EtOH exposure, long term EtOH over-exposure prevents stimulation of CBF, reduces the response of cAMP-dependent protein kinase (PKA) and cGMP-dependent protein kinase (PKG) to their respective agonists, and lowers nitric oxide (NO) concentration in the bronchial lavage fluid (BAL) in mouse models [23].

CBF depends on the phosphorylation of axonemal proteins on the beta and gamma chains of the outer dynein arm of the cilia. EtOH exposure alters the phosphorylation of axonemal proteins on the outer dynein arm, and their phosphorylation corresponds with AICD [26]. Price *et al.*, revealed that exposure to EtOH activates protein phosphatase 1 (PP1), and blocks cAMP dependent ciliary beating and PKA responsiveness [19]. However, exposure to a PP1 inhibitor restored the responsiveness of the protein kinase and reversed the inhibition of cAMP dependent ciliary beating, identifying PP1 as a potential therapeutic target [19]. Subsequently, the Sisson group demonstrated EtOH-treated mice that received dietary supplements of the antioxidants N-acetylcysteine (NAC) and procysteine were able to retained their ciliary function, however, no subsequent increase in nitric oxide levels were observed [23].

Collectively, chronic EtOH-mediated reduction in CBF allows more pathogens to bypass this first line of defense in the lungs resulting in impaired pulmonary immune response against infections and the development of pathological lung diseases.

## 3. EtOH Impairs Pulmonary Innate Immune Response

Chronic EtOH exposure results in ROS impaired alveolar macrophages (AMs), harming the pulmonary immune response and increasing the likelihood of bacterial infection and pneumonia. For this section of the review we will mainly focus on the AMs since these cells play an intricate role in the pulmonary innate immune response.

### 3.1. AMs, NADPH Oxidases and EtOH Exposure

Major sources of intrinsic oxidants from AMs are the NADPH oxidases, also known as the NOX family of enzymes [27]. NOX enzymes, including NOX 1–5, Duox 1, and Duox 2, are transmembrane proteins that catalyze the transfer of electrons across the membrane, producing superoxide radicals that rapidly dismutates into hydrogen peroxide [28,29]. Evidence has shown that EtOH directly up regulates the NADPH oxidases NOX 1 and NOX 2, which leads to downstream activation of NOX 4 and increased oxidative stress [30]. Furthermore, mitochondrial oxidative stress impairs redox signaling in AMs, thus limiting phagocytosis, a key function of AMs in removing pathogens and debris [31]. Liang *et al.* observed that AMs exposed to EtOH resulted in NADPH and glutathione (GSH) depletion, oxidation of the thioredoxin circuit, and hyperoxidation of mitochondrial peroxiredoxins [32]. Chronic exposure of EtOH also decreased expression of thioredoxin 2, thioredoxin 2 reductase, and peroxiredoxins 3 and 5, all of which are crucial elements of the thioredoxin circuit [33]. This depletes the antioxidant capacity of the cells and compromises the cell’s ability to detoxify ROS and inhibits phagocytosis [32]. Consequently, this inhibition of AMs phagocytosis, decreased by as much as 50%, can severely impair the initial immune response [34].

### 3.2. EtOH Regulates TGF-β Expression

The nuclear factor erythroid 2 (NFE2)-related factor 2 (Nrf2), a redox sensitive transcription factor, is another important element within the lungs that responds to changes in the oxidant/antioxidant balance. Nrf2 regulates the antioxidant response element, however, chronic exposure to EtOH down regulates Nrf2, which consequently decreases antioxidant response element, decreases expression of antioxidant genes, and reduces intracellular glutathione (GSH). Jensen *et al.* showed that these changes were reversed in EtOH-exposed cells also treated with an Nrf2 activator [35]. Increased transforming growth factor beta (TGF-β) suppresses the activity of Nrf2, which in turn inhibits the antioxidant response element [36]. The antioxidant response element is a signaling pathway that activates genes necessary for eliminating ROS, is crucial in combatting oxidative stress [37]. Conversely, a study of alveolar barrier function in HIV-1 models demonstrated activating Nrf2 restores the epithelial barrier function, although this has not been investigated in cells exposed to EtOH [38].

In addition to oxidative stress, chronic EtOH exposure also induces TGF-β expression and leads to alternative activation in AMs, along with decreasing mitochondrial membrane potential and adenosine triphosphate (ATP) [39,40]. GSH supplementation and exposure to TGF-β antagonists were both shown to reverse the effects of EtOH on mitochondrial oxidative stress, TGF-β production, and phagocytosis in mouse models [40,41]. Furthermore, treatment with the antioxidant mitoTEMPOL, a piperidine nitroxide that targets the mitochondria and protects against lipid peroxidation as well as DNA damage, reversed the impaired phagocytosis and cell proliferation associated with chronic EtOH exposure [39,42].

### 3.3. EtOH Impairs AM Function via Zinc Deficiency

As well as impairing phagocytosis through TGF-β and NADPH oxidase activity, over-exposure to EtOH also impairs AM function by modulating zinc uptake [43]. Zinc is an essential cofactor for thousands of transcription factors and enzymes [44]. Chronic EtOH exposure leads to decreases intracellular zinc concentration in AMs, although the serum concentration of zinc is unchanged [45]. EtOH has been shown to decrease expression of Kruppel-like transcription factor 4 (KLF4), a zinc-finger containing transcription factor that regulate AMs polarization, thereby impairing the response of alveolar macrophages to pathogens and reducing phagocytosis [46]. In addition, KLF4 binds the transmembrane zinc transporter ZIP4, which imports zinc from blood serum [46]. Expression of ZIP4 is likewise inhibited in alveolar epithelial cells and AMs chronically exposed to EtOH [47]. This decrease in both KLF4 and ZIP4 expression results in a drastic reduction in zinc transport, which further impairs the phagocytic function of AMs and their responsiveness to pathogens [46,47].

Granulocyte/macrophage colony stimulating factor (GM-CSF) is a secreted peptide essential for the differentiation of circulating monocytes into alveolar macrophages via the transcription factor PU.1 [48]. In the absence of GM-CSF, AMs show reduced phagocytosis and pathogen killing [48]. Joshi *et al.* showed that chronic EtOH exposure in rodent models led to decreased membrane expression of the GM-CSF receptor and reduced PU.1 expression in AMs [49]. In EtOH-fed rats, dietary zinc restored PU.1 nuclear binding in AMs, increased Nrf2 nuclear binding, and normalized bacterial clearance [50]. These studies suggest that EtOH impairs the phagocytic capabilities of AMs via zinc deficiency, which further impairs the innate immune response.

### 3.4. EtOH Impairs Ability of AMs to Bind Pathogens

In addition to impairing phagocytosis, exposure to EtOH can also weaken AMs ability to initially bind invading pathogens. Asplund *et al.*, revealed that when the murine macrophage-like cell line J774.16 was exposed to EtOH, they lose their ability to bind *Acinetobacter baumannii*, a pathogen that commonly causes community-acquired pneumonia [34]. The binding ability was reduced by more than 50% as compared to control cells and this loss was associated with decreases in the GTPase RhoA, which has previously been identified as a key component of phagocytosis [51]. This loss of RhoA consequently results in decreased actin polymerization which, further impairs the formation of the phagocytic pocket [34]. Conversely, in many other systems, EtOH has been shown to up regulate RhoA expression. Cultured astrocytes chronically exposed to EtOH had increased RhoA expression and disorganized actin cytoskeleton [52]. Additionally, several studies have shown that chronic exposure to EtOH up regulates RhoA expression in the intestinal epithelium and leads to decreased barrier function [53,54]. Taken together, these studies imply that altered RhoA expression plays an important role in EtOH-mediated dysfunction.

## 4. EtOH Impairs the Adaptive Immune Response

EtOH over-exposure alters the adaptive immune response to pathogens, thereby increasing the risk of developing an infectious lung disease. In addition to increasing the risk of developing pneumonia eightfold, EtOH exposure has also been shown to be a risk factor for infection with *Mycobacterium tuberculosis* [55]. Mason *et al.* showed that the bacterial burden in the lungs of EtOH-fed mice was significantly higher than that of mice fed a control diet [56]. The increased bacterial burden was associated with a decrease in CD4^+^ and CD8^+^ lymphocytes and reduced lymphocyte proliferation [56].

The cytokine interferon-γ (IFN-γ), is critical to limiting bacterial infection in the lung [57]. EtOH has been shown to impair signaling by IFN-γ, thus reducing the immune response to pathogens [58]. In mouse models of tuberculosis, EtOH-fed mice showed both a decreased CD4^+^ response and a decreased IFN-γ response [58]. Similarly, in animal models of chronic EtOH exposure, Gurung *et al.* revealed that the primary CD8^+^ T-cell response was impaired, both antigen-specific CD8+ T-cells and memory cell numbers were reduced, and lymphocyte proliferation was reduced after inoculation with *Listeria monocytogenes* [59].

Dendritic cells are also important in the adaptive immune response. These leukocytes are responsible for the processing and presentation of antigens to T-cells, functions which are impaired when cells are chronically exposed to EtOH [60]. Chronic EtOH exposure was shown to reduce the ability of CD11C+ dendritic cells to support an IFN-γ response to CD4^+^ cells. Dendritic cell numbers were also shown to be reduced in the spleen and increased in the thymus of EtOH-fed mice as compared to the control, further implicating dendritic cells in the EtOH-induced impaired immune response [61].

Furthermore, cocultures of CD4^+^ and antigen-presenting cells showed alterations in pro-and anti-inflammatory cytokines associated with EtOH exposure. In chronic EtOH exposure, cells showed decreased levels of interleukin (IL)-6, IL-12, IL-17A, and IFN-γ, along with increased IL-13 in response to stimulation, as compared to controls [61]. IL-17, produced by CD4^+^ and CD8^+^ T cells, up regulates the expression of cytokines and chemokines that promote neutrophilic inflammation [62,63]. EtOH exposure suppresses IL-17 release, decreases neutrophil recruitment, and increases mortality from *Klebsiella pneumoniae*. In animal studies, treatment with an IL-17 encoding adenoviral vector was shown to improve survival of *K. pneumoniae* infection [62].

The lungs of EtOH-fed mice also showed reduced expression of Il-12, with an increase in IL-10 mRNA and protein, as compared to controls. This led to suppressed bacterial clearance and decreased survival of infection with *K. pneumoniae* [64]. However, neutralization of IL-10 resulted in decreased lung bacteria and increased survival [65].

EtOH affects the function of the adaptive immune response through multiple pathways, acting on dendritic cells, T cells, and cytokine signaling, collectively weakening the immune response to pathogens and increasing susceptibility to infection.

## 5. EtOH Alters Airway Epithelial Permeability

### 5.1. EtOH Dysregulates Ion Transporters

A hallmark of EtOH-induced lung dysfunction, particularly in ALI, is increased alveolar epithelial permeability and reduced fluid clearance resulting in pulmonary edema [66,67,68]. Although EtOH alone does not cause ALI, it increases the risk of developing ALI in patients with sepsis and trauma [70]. Chronic over-exposure to EtOH increases the risk of developing ALI twofold, while also increasing severity and mortality of the disease [69,70]. In a normal lung, sodium (Na^+^), chloride (Cl^−^), and potassium (K^+^) ions are transported by epithelial sodium channels (ENaC) and Na,K-ATPase which regulate the clearance of fluid from the alveolar space, resolving pulmonary edema [71]. ENaCs reabsorb Na^+^ from the airway lumen and transport it into the cytoplasm, where it is extruded with an ATPase pump to build an electrochemical gradient and facilitate water reabsorption [72]. EtOH-induced leakage in the epithelium leads to impaired Na^+^ and Cl^−^ transport, resulting in increased fluid in the alveolar space and pulmonary edema. Furthermore, the interplay of ENaC, NADPH oxidases, and TGF-β are increasingly implicated in the development of EtOH-induced lung injury [73].

In chronic EtOH exposure, the up-regulation of NOX 4 mediated ROS modulates ENaC protein activity [30,74]. In addition, studies conducted by Downs *et al.* demonstrated that EtOH exposure also modified the cysteine residues of α-ENaC proteins and increased ENaC activity in lung alveolar cells, leading to increased fluid clearance and a potentially pro-injury environment in the lungs [75]. The Downs group also demonstrated EtOH-induced TGF-β signaling occurred through Tgfbr1 and Smad2/3, recruitment of NADPH oxidases, and the subsequent generation of ROS [75]. This led to the internalization of the αβγENaC complex and the inhibition of sodium transport and fluid reabsorption [73].

### 5.2. EtOH Disrupts Barrier Function

An important regulator of alveolar epithelial barrier function is the tight junction, which anchors epithelial cells together and prevents the influx of fluid into the airspace [76]. Transmembrane claudins are essential to the function of tight junctions; in the lungs, claudin-3, claudin-4, and claudin-18 are most highly expressed. Disrupting claudin homeostasis via EtOH exposure therefore primes the lungs for the development of pulmonary edema [76]. Recent studies of tight junctions in the intestinal epithelium suggest an important role for RhoA in EtOH-induced epithelial leakage, as well as a role in EtOH-induced impaired phagocytosis. Exposure to EtOH induces the expression of RhoA, a GTPase involved in actin filament reorganization [34]. Increased RhoA expression induces phosphorylation of myosin light chains resulting in the disassembling of tight junction [77]. Furthermore, the inhibition of RhoA expression by shRNA restored transepithelial resistance and inhibited epithelial leakage [77].

Curry-McCoy *et al.* demonstrated that the interplay between AMs and the lung epithelium is also important to maintaining epithelial barrier function [47]. In their study of the effects of EtOH on membrane permeability, AMs were exposed to EtOH to increase membrane-bound TGF-β. Alveolar epithelial cells treated with cell lysate from these AMs revealed decreased epithelial barrier function, which was alleviated by treatment with an anti-TGF-β antibody [47] Additionally, a second study by Overgaard *et al.* found that in alveolar epithelial cells, treatment with TGF-β1 decreased transepithelial permeability by 25% [78]. Collectively, EtOH exposure reduces the clearance of fluid from the lungs and increases epithelial permeability, priming the lung for the development of acute lung injury.

## 6. EtOH Exposure Is Implicated in Matrix Remodeling

Fibrosis in the liver has been linked to chronic EtOH over-exposure and subsequent TGF-β1 activation, although the role of EtOH in lung fibrosis is less clear [79]. However, chronic EtOH exposure has been linked to decreased levels of glutathione and increased expression of TGF-β1, both of which are implicated in pulmonary fibrosis [80,81,82,83]. Fibrosis is characterized by excessive deposition of the extracellular matrix (ECM) [84]. Chronic EtOH exposure has been shown to induce alterations in tissue remodeling and the ECM in lung tissue, including an increase in expression of the matrix protein fibronectin [85].

Sueblinvong *et al.*, showed that in mice models of bleomycin-induced ALI, EtOH-fed mice showed fibrosis, a 75% increase in lung collagen deposition, and a 120% increase in TGF-β1 in BAL fluid 14 days after bleomycin installation. However, supplementation with s-adenosylmethione (SAMe a glutathione precursor) attenuated these effects in EtOH-fed mice by decreasing TGF-β1 expression [86]. This further implicates EtOH in matrix remodeling and the pathogenesis of fibrosis.

EtOH has also been shown to activate matrix metalloproteinases (MMPs) in the lungs, particularly MMP-2 and MMP-9 [87]. MMP-9, a type IV collagenase, is present at low levels in healthy lung tissue, but is elevated in idiopathic pulmonary fibrosis (IPF), COPD, and asthma [88]. EtOH-induced MMP activation leads to the disruption and degradation of the alveolar extracellular matrix, a key feature of ALI [87].

## 7. Conclusions

EtOH has multiple deleterious effects on the lungs, many of which we are only beginning to understand. EtOH contributes to lung dysfunction by altering barrier function in the airway epithelium, impairing the binding and phagocytic abilities of AMs, and deregulating mucociliary clearance in the airway. EtOH exposure leads to decreased clearance of pathogens through several pathways, including a decrease in CBF via PP1 activation and impaired AM function through upregulation of NOX and TGF-β and down regulation of zinc transporters. EtOH acts on the adaptive immune system as well, via dendritic cells, T cells, and signaling cytokines. This combination of impaired CBF and a weakened immune response leads to the pathogenesis of infectious lung disease, including the bacterial pneumonia common among heavy drinkers. Additionally, EtOH-mediated epithelial permeability and impaired fluid clearance via ion channel dysregulation and increased TGF-β predispose the lung to ALI, and EtOH-induced matrix remodeling suggests a role for EtOH in lung fibrosis. A thorough understanding of the effects of EtOH and its metabolites on gene expression, cell signaling, and oxidative stress will lead to better treatment options for EtOH-induced lung disease, which affects thousands of people in the United States.
